# Febrile Immunocompromised Renal Transplant Recipient with Allograft Dysfunction: Detection of an Undiagnosed Prostate Abscess by [ ^18^ F]FDG-PET/CT along with Treatment Response Monitoring

**DOI:** 10.1055/s-0044-1786705

**Published:** 2024-05-07

**Authors:** Sunita Nitin Sonavane, Tukaram Jamale, Sreyasi Bose, Sandip Basu

**Affiliations:** 1 Radiation Medicine Centre, Bhabha Atomic Research Centre, Tata Memorial Hospital Annexe, Parel, Mumbai, Maharashtra, India; 2 Homi Bhabha National Institute, Mumbai, Maharashtra, India; 3 Department of Nephrology and Transplant, King Edward Memorial Hospital and Seth GS Medical College, Parel, Mumbai, Maharashtra, India

**Keywords:** prostatic abscess, infection, renal transplant, febrile, [ ^18^ F]FDG PET-CT, follow-up

## Abstract

The purpose of this report is to provide a comprehensive description of a post-transplant febrile patient's clinical course, complications, surgical procedure, and long-term management including evaluation by ^18^ F-fluorodeoxyglucose [( ^18^ F)FDG] positron-emission tomography combined with computed tomography (PET/CT). A 35-year-old male, a postrenal transplant patient, developed chronic allograft dysfunction and presented with fever with chills, with suspicion of acute-on-chronic graft dysfunction, but no infective focus localization on chest X-ray, ultrasonography (USG) whole abdomen, or blood culture. Urine microscopy showed 8 to 10 pus cells/high-power field (hpf) and culture showed *Klebsiella pneumoniae* and *Pseudomonas aeruginosa* with low colony count. Culture-sensitive antibiotics were prescribed for 2 weeks, and after 3 weeks febrile episodes relapsed, symptoms progressed, and required emergency hospitalization due to acute painful urinary retention. Proteinuria and no growth were noted in urine analysis, serum creatinine was 5.36 mg/dL, and C-reactive protein was 15.7mg/dL, and remaining parameters were unremarkable. [ ^18^ F]FDG-PET/CT was considered in order to resolve diagnosis, which revealed abnormal heterogeneous tracer uptake in the enlarged prostate with hypodense areas within, suggesting prostatitis with abscess formation and pyelonephritis in the upper pole of the transplant kidney. USG kidney urinary bladder (KUB) correlation confirmed prostatic abscess and transurethral drainage done, and pus culture revealed *Burkholderia pseudomallei* . Culture-sensitive intravenous meropenem treatment was given for 3 weeks. At 5 weeks, follow-up [ ^18^ F]FDG-PET/CT showed low metabolic residual prostate uptake, suggesting a good response with residual infection. Thus, intravenous antibiotics was changed to oral antibiotics for another 6 weeks. His symptoms completely resolved at the end of treatment; however, his graft function worsened, with serum creatinine reaching 6 to 7 mg/dL, and eventually, after 8 months he became dialysis dependent.

## Introduction

Atypical or subtle presenting symptoms pose complex challenges in febrile immunocompromised renal transplant recipients, wherein ^18^ F-fluorodeoxyglucose positron-emission tomography-computed tomography ([ ^18^ F]FDG PET/CT) can aid as an initial noninvasive diagnostic modality for earlydiagnosis, 1 directing prompt management, and thereby playing a crucial role in preventing further potential complications and optimizing graft salvation. Acute bacterial prostatitis presents with urinary tract symptoms and pelvic pain in men. 2 It comprises up to 10% of all prostatitis diagnoses, and its incidence peaks at 20 to 40 years and older than 70 years of age.

Men at high risk of prostate infection include those with significant chronic medical conditions such as poorly controlled diabetes, end-stage renal disease patients on hemodialysis, liver cirrhosis, cancer patients receiving chemotherapy, transplant recipients, human immunodeficiency virus/acquired immunodeficiency syndrome patients, men with inadequately treated benign prostatic hyperplasia, patients with chronic foley or suprapubic catheters, and neurogenic bladder dysfunction 3 and others with immunodeficiency. 3 One of the feared complications of acute bacterial prostatitis is a prostatic abscess. 4

This case highlights the usefulness of [ ^18^ F]FDG-PET/CT in localizing the cause of fever when no localized signs are available in immunocompromised status, aiding in localization, characterization, and post-treatment response monitoring to ensure complete resolution of the infection focus, thereby deciding antibiotic strategy and salvaging transplant function.

## Case Report

**Fig. 1:**
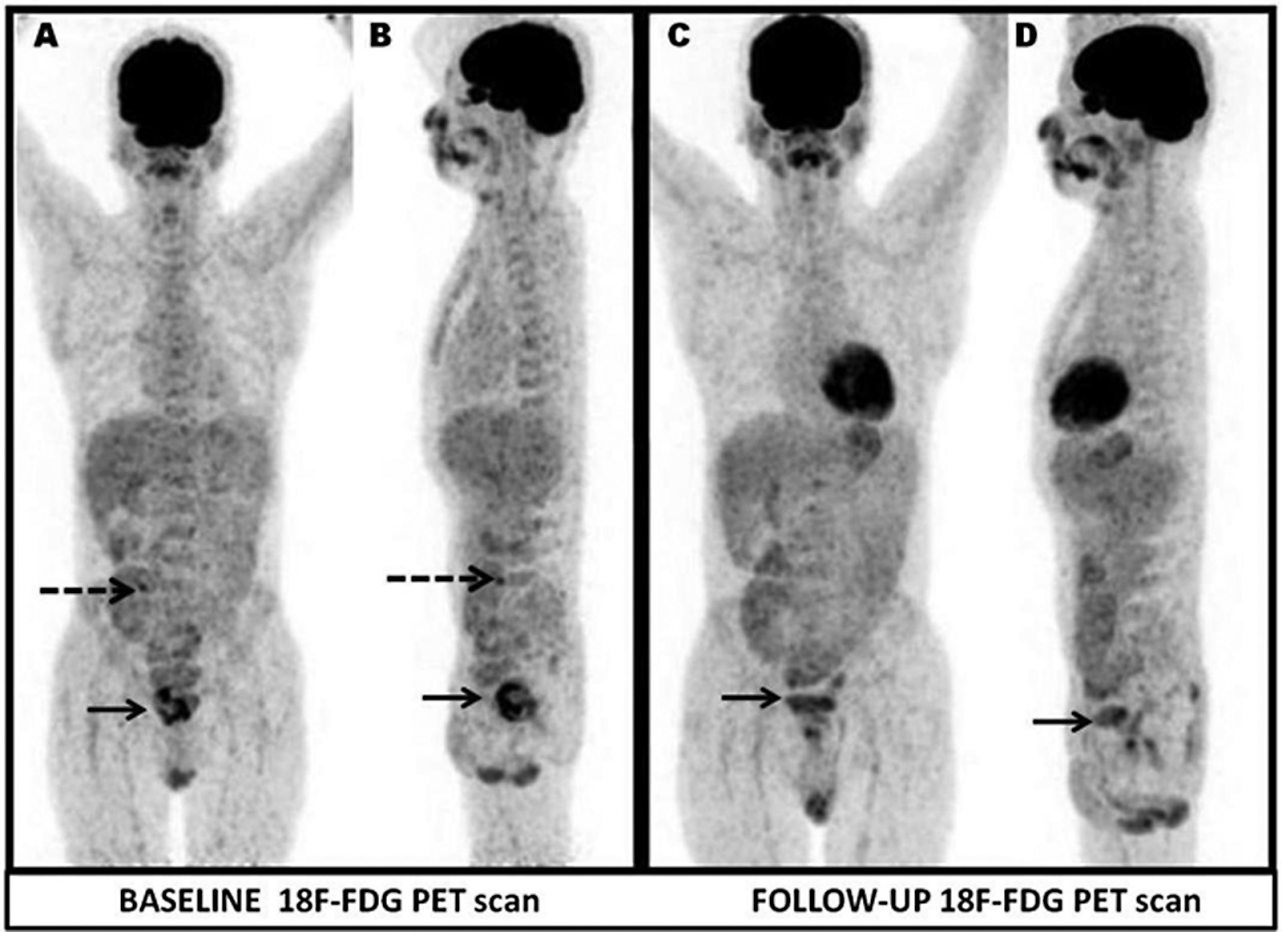
^18^ F-fluorodeoxyglucose positron emission tomography (18F-FDG PET) whole-body maximal intensity projection images. Anterior ( **A, C** ) and left lateral ( **B,D** ) and baseline ( **A, B** ) images showing abnormal heterogenous tracer uptake in enlarged prostate (maximum standardized uptake value [SUVmax]: 6.85) with relative cold areas within metabolically active lesions (black thick arrow), focal tracer uptake (SUVmax: 4.75, white dotted arrow) in upper pole of transplant kidney in right iliac fossa; Follow-up ( **C, D** ) showing no new lesion, residual low-grade tracer uptake in the prostate (SUVmax: 3.57, black thick arrow), transplant renal cortex unremarkable.

**Fig. 2:**
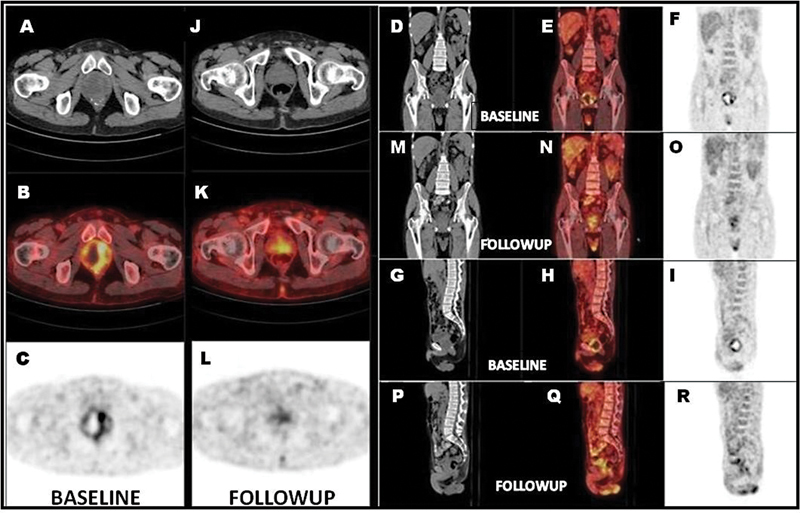
18F-fluorodeoxyglucose positron emission tomography (18F-FDG PET) whole-body maximal intensity projection images. Anterior (A, C) and left lateral (B,D) and baseline (A,B) images showing abnormal heterogenous tracer uptake in enlarged prostate (maximum standardized uptake value - 6.85) with relative cold areas within metabolically active lesions (black thick arrow), focal tracer uptake (SUVmax: 4.75; white dotted arrow) in upper pole of transplant kidney in right iliac fossa; Follow-up (C, D) showing no new lesion, residual low-grade tracer uptake in the prostate (SUVmax: 3.57; black thick arrow), transplant renal cortex unremarkable.

We herein present a 35-year-old male, liver-renal transplant recipient (maternal donor); 2 years post-transplant was started on dual immunosuppressants (steroids and mycophenolate Mofetyl); after 15 years he was diagnosed with chronic allograft dysfunction having baseline serum creatinine levels ranging from 3 to 4 mg/dL. He presented with fever episodes, associated with chills, acute on chronic graft dysfunction (serum creatinine: 4–5 mg/dL); however, there was no localization of the infective focus on chest X-ray, USG abdomen, and blood culture was normal. Urine routine microscopy showed 8 to 10 pus cells/high-power field (hpf). Urine culture on two separate occasions was positive for *Klebsiella pneumoniae* and *Pseudomonas aeruginosa* with low colony count. Based on culture sensitivity report, the patient was treated with oral levofloxacin for 2 weeks. Following this treatment, the patient became afebrile for 3 weeks, presented with recurrence of intermittent high-grade febrile episodes accompanied by dysuria and a poor urinary stream. Symptoms progressed in a few days to acute painful urinary retention that required emergency hospitalization. Urine analysis revealed pus cells pf 1 to 2/hpf, red blood cells of 8 to 10/hpf, protein ++, no growth in culture, serum creatinine of 5.36 mg/dL, and C-reactive protein of 15.7 mg/dl, while the remaining blood parameters were unremarkable. The diagnosis was unresolved, and [ ^18^ F]FDG-PET/CT was performed given the persistent fever despite treatment with antibiotics as per urine culture sensitivity, suspecting pyelonephritis. [ ^18^ F]FDG-PET/CT revealed abnormal heterogeneous tracer uptake in enlarged prostate (volume: 76cc, maximum standardized uptake value [SUVmax]: 6.85) with hypodense areas within suggesting infectious etiology-prostatitis with abscess formation and focal [ ^18^ F]FDG uptake noted in upper pole of transplant kidney in right iliac fossa (SUVmax:4.75) denoting pyelonephritic involvement ( Figs. 1 and 2 ). Subsequent correlation with USG KUB revealed bulky prostate with hypoechoic collection, suggesting prostatic abscess and transplant kidney was unremarkable. The patient underwent transurethral drainage of prostatic abscess, and pus culture revealed the growth of *Burkholderia pseudomallei* and was negative for tuberculosis. Based on pus culture and sensitivity report, the patient was advised to take intravenous meropenem for 3 weeks. A follow-up [ ^18^ F]FDG-PET/CT was considered to look for a resolution of the abscess and decide on the duration of antibiotics. The follow-up PET-CT at 5 weeks showed no new lesion, residual ill-defined hypodense lesion with low-grade [ ^18^ F]FDG uptake in the prostate (SUVmax: 3.57), which had significantly reduced in size and uptake suggesting a good response to treatment with residual infection ( Figs. 1 and 2 ). Thus, intravenous meropenem was stopped and converted to oral sulfamethoxazole and trimethoprim single-strength twice daily for 6 weeks on discharge.

His symptoms had completely resolved at the end of treatment; however, his graft function had worsened compared to baseline during the course of the illness. The serum creatinine at the end of treatment remained in the range of 6 to 7mg/dL. He was closely followed up, and 8 months after a second [ ^18^ F]FDG scan, eventually he was initiated on dialysis because of uremic complaints.

## Discussion

Prostatic abscesses are relatively rare; immunocompromised individuals are more susceptible and typically present with nonspecific symptoms, which pose diagnostic challenges resulting in delayed diagnosis and late treatment, causing the morbidity and mortality of this condition. 5 A high level of clinical suspicion, particularly in high-risk and nonresponding individuals, is required for early diagnosis, timely treatment, and optimal outcomes. 6 Elshal et al reported 42 patients of prostatic abscess who were admitted for active interventional treatment of whom 24% were on immunosuppressive treatment secondary to a renal/liver transplant. 7

In this case, [ ^18^ F]FDG uptake in prostate led to timely diagnosis. As we know accurate diagnosis using tools such as [ ^18^ F]FDG-PET/CT imaging can aid in early identification of infection, allowing prompt initiation of appropriate treatment, immunosuppressive therapy, infection prophylaxis, and long-term graft outcomes. 6 Kang et al evaluated the clinical value of incidental prostate uptake on ^18^ F-FDG-PET/CT by selecting cases that reported focal/diffuse FDG uptake in the prostate. Further analysis revealed, no significant correlation between scan findings and serum prostate-specific antigen (PSA) levels and SUVmax in all patients. Median SUVmax was higher in the prostate cancer group (mean: 10.1, range: 3.8–24.5) than in the benign group (mean: 4.3, range: 3.1–8.8), but the difference was not statistically significant. Incidental prostate uptake on ^18^ F-FDG-PET/CT scans should not be ignored and should undergo further clinical evaluations, such as PSA and digital rectal examination. 8

Kao et al detected diffuse ^18^ F-FDG uptake in biopsy proven prostatitis. 9 Jadvar highlighted the rarity of incidental high FDG uptake in the prostate gland, prompting for further investigation with at least a measurement of serum PSA level. They further mentioned FDG uptake level may significantly overlap among normal, benign, and malignant tissues; aggressive primary tumors with Gleason score more than 7 tend to display higher FDG uptake. 10 A multivariate analysis indicated that the presence of bacteria in an expressed prostatic secretion increased the risk of incidental prostate ^18^ F-FDG uptake in nonmalignant prostate tissues. 11

The study by Kim et al showed that in bacillus Calmette–Guérin (BCG)-induced granulomatous prostatitis, focal or multifocal prostate uptake is frequently seen within 1 year after BCG therapy, and the intensity of prostate uptake is decreased on the follow-up PET/CT without any treatment. 12 Additionally, regular follow-up scans and close clinical monitoring are crucial in these patients to detect and manage complications such as abscess formation. The literature has also documented the utility of [ ^18^ F]FDG-PET/CT imaging in diagnosing and guiding the treatment of prostate infection in a renal transplant patient. 13 CT improves radiological assessment of the prostate and documentation of the role of [ ^18^ F]FDG-PET/CT in prostate infection is reported in isolated reports. 14

Patients treated for acute bacterial prostatitis who fail to improve after 48 hours, especially high-risk immunocompromised individuals, should be promptly evaluated for a prostatic abscess. Abscesses smaller than 2 cm in diameter can be treated conservatively with culture-specific antibiotics but will respond more quickly if the abscess is aspirated and drained; this treatment policy was used to manage the present case. Serial follow-up imaging studies and urine culture examinations are recommended to ensure complete resolution of the abscess. 6 If locoregionally not controlled, systemic spread of infection can occur in severe cases and prostate infections can become chronic or recurrent, posing challenges in management; 15 thus, delicate balance may be necessary while treating infections in immunocompromised individuals, encompassing adjustments to immunosuppressive medications to control the infection while minimizing the risk of organ rejection.

Prostate abscesses management may require drainage and definitive microbiological documentation guiding antimicrobial selection. 16 Incorrect diagnosis and delayed management may result in untoward graft-related adverse outcomes, which can potentially affect the function of the transplanted kidney, 17 rarely death in severe cases. 16 Our case patient underwent transurethral drainage of prostatic abscess, and pus culture revealed the growth of *Burkholderia pseudomallei* . *Burkholderia pseudomallei* , the environmental bacterium responsible for the disease melioidosis, is endemic in northern Australia and southeast Asia. 18

In summary, [ ^18^ F]FDG-PET/CT played a vital role in diagnosing and guiding the treatment of prostate infection in a renal transplant patient. The report discussed the varying challenges and possibilities encountered in the management of renal transplant recipients, with focus on early detection of infective focus by [ ^18^ F]FDG-PET/CT, in conjunction with prompt initiation and response monitoring of appropriate treatment.

## Conclusion

[ ^18^ F]FDG-PET/CT scan aid in unveiling the cause of fever when no localizing signs are noted in immunocompromised patients; it aids in infection localization, characterization, and in post-treatment response monitoring by demonstration of reduction or resolution of the infection focus, thereby deciding the time to convert from intravenous to oral antibiotics, and further time to discontinue antibiotics when scan negative.
